# Humidity- and Temperature-Sensing Properties of 2D-Layered Tungsten Di-Selenide (2H-WSe_2_) Electroconductive Coatings for Cotton-Based Smart Textiles

**DOI:** 10.3390/polym17060752

**Published:** 2025-03-12

**Authors:** Valentina Trovato, Rajashree Konar, Eti Teblum, Paolo Lazzaroni, Valerio Re, Giuseppe Rosace, Gilbert Daniel Nessim

**Affiliations:** 1 Department of Engineering and Applied Sciences, University of Bergamo, Viale Marconi 5, 24044 Dalmine, Italy; paolo.lazzaroni@unibg.it (P.L.); valerio.re@unibg.it (V.R.); giuseppe.rosace@unibg.it (G.R.); 2 Department of Chemistry, Bar-Ilan University, Ramat-Gan 5290002, Israel; rajashreekonar@gmail.com (R.K.); eti.teblum@biu.ac.il (E.T.); 3 Institute of Nanotechnology & Advanced Materials (BINA), Bar-Ilan University, Ramat-Gan 5290002, Israel; 4 International Iberian Nanotechnology Laboratory, Av. Mte. José Veiga, 4715-330 Braga, Portugal

**Keywords:** transition metal dichalcogenides, 2D materials, tungsten di-selenide, smart textiles, wearable sensors, textile finishing, electroconductive coatings, environmental monitoring

## Abstract

Electroconductive textiles (e-Textiles) are vital in developing wearable sensors that preserve the comfort and characteristics of textiles. Among two-dimensional (2D) transition metal dichalcogenides (TMDs), considered a promising option for sensor applications, tungsten di-selenide (WSe_2_) homostructures have been used as humidity- and temperature-sensing materials for developing e-textiles, as mentioned in a first-of-its-kind report. Exfoliated chemical vapor deposition (CVD)-grown 2H-WSe_2_ nanosheets were dispersed in hydroalcoholic solutions using an amino-functionalized silane to improve dispersion. Acrylic thickener was added to create 2H-WSe_2_-based pastes, which were applied onto cotton using the knife-over-roll technique to obtain thin, flexible electroconductive coatings on textiles. Various characterization techniques confirmed the even distribution of 2D-WSe_2_-based coatings on fabrics and the maintenance of textile comfort and wearability. The conductivity of coated fabrics was measured at room temperature and ranged between 2.9 × 10^8^ and 1.6 × 10^9^ Ω sq^−1^. The WSe_2_-based textile sensors functioned well as resistance humidity detectors within 30–90% relative humidity (RH), revealing good repeatability and sensitivity after multiple exposure cycles. To a lesser extent, WSe_2_-based textile sensors act as temperature detectors within 20–60 °C with limited repeatability. The 2D-based textiles exhibited a quadratic dependence of resistance on temperature and a characteristic thermal hysteresis. This proposed strategy marks a significant milestone in developing scalable and flexible 2D TMD-based detectors with great potential for wearable sensing devices.

## 1. Introduction

Environmental humidity and temperature monitoring are critical for diverse sectors, including healthcare, agriculture, industrial processing, and environmental surveillance. Since fluctuations in these parameters can significantly affect biological processes, product quality, and system efficiencies, there is a pressing need for reliable and sensitive sensors to ensure adequate environmental management.

While numerous materials have been recently explored for humidity and temperature sensing [1,2,3,4], the fascinating optical and electrical properties of two-dimensional (2D) materials, particularly transition metal dichalcogenides (TMDs), make them increasingly popular for electronic device applications [5,6,7]. Furthermore, they are promising for next-generation nanoelectronics due to their tunable bandgap based on the size and charge of the elements [8].

TMDs can also be a great alternative to carbon nanotubes (CNTs) or graphene for simple and low-cost preparation of intelligent textiles, which are helpful in flexible electronics [9]. Encouraging experimental results on graphene and its derivatives (e.g., graphene oxide and reduced graphene oxide) [10], as well as black phosphorus [11], have demonstrated their suitability as humidity-sensing materials. These findings have driven scientific research toward exploring transition metal dichalcogenides (TMDs) for detecting inorganic species in chemoresistive humidity sensors [12,13,14,15].

TMDs, such as molybdenum disulfide (MoS_2_), molybdenum diselenide (MoSe_2_), tungsten disulfide (WS_2_), and tungsten diselenide (WSe_2_), offer enhanced sensitivity and selectivity due to their high surface area-to-volume ratios and layered structures, which facilitate the adsorption and desorption of water molecules, resulting in significant variations in electrical resistance or capacitance [16]. Moreover, unlike CNTs and graphene, whose electronic properties are fixed, TMDs can exhibit semiconducting, metallic, or ambipolar behavior depending on their composition and layer thickness, thereby ensuring a high sensor response [17]. In addition, materials like WSe_2_ minimize heat dissipation due to their low thermal conductivity, making them particularly promising for temperature monitoring [17]. TMDs also demonstrate superior chemical stability compared to graphene, which is beneficial under fluctuating environmental conditions, and they operate efficiently at room temperature, unlike many metal oxide-based sensors, rendering them suitable for low-power, wearable applications [18]. For instance, WS_2_/WSe_2_ nanohybrids produced via liquid-phase co-exfoliation have shown remarkable stability, reproducibility, and minimal hysteresis in humidity detection [13]. Similarly, WSe_2_ nanosheets obtained through sonochemical exfoliation have exhibited high sensitivity and responsiveness to human breathing and touchless humidity modulation, underscoring their potential in advanced biomedical and intelligent electronic applications [19].

Commercial thermistors, by contrast, often suffer from limited operating ranges, high nonlinearity, drift, and noise, which emphasize the need for novel materials that deliver improved sensitivity, stability, and operational efficiency. TMDs have been widely investigated for temperature-sensing applications due to their anisotropic charge carrier, phonon transport, and exceptional thermal properties. Thermistors fabricated using MoSe_2_ via drop-casting, for example, exhibit highly linear temperature-dependent electrical responses, stable semiconducting behavior, excellent reproducibility, and negligible thermal hysteresis. These devices have demonstrated a temperature coefficient of resistance (TCR) of approximately −0.51%/K in the 273–373 K range, increasing to about −1.75%/K at lower temperatures (273–173 K) [20].

While MoS_2_ is a popular choice for 2D TMDs, tungsten-based dichalcogenides are gaining attention due to the larger size of the W atom. Additionally, its commercial viability and greater availability in mineral resources make tungsten-based dichalcogenides a promising candidate for future industrial applications [21]. Indeed, especially WS_2_, these are fascinating materials for temperature sensing thanks to their high charge carrier mobilities, ease of exfoliation into a few or monolayers, optimal bandgaps, and in-plane heat transportation (similar to other 2D materials, like graphene) [22]. In a research study [23], WS_2_ nanoflakes were used for developing an electrochemical power generation humidity sensor without adding additional alkali metal salts, that exhibits a wide humidity detection range (18.7–91.5% relative humidity), fast response/recovery times (8.4/5.2 s), good repeatability (20 cycles), and small humidity hysteresis (~3.4% RH) at room temperature of 25 °C. Although WS_2_ is still the most frequently used molecule among TMDs in various optoelectronic applications [24,25], tungsten di-selenide (WSe_2_) has attracted research interest for future wearable electronic and optoelectronic devices due to its superior physical, optical, and electrical properties [26]. It is a p-type inorganic 2D material with high hole mobility, characterized by W atoms confined in a trigonal prismatic coordination sphere neighbored by Se atoms. It has been recently investigated for numerous promising applications, such as field-effect transistors (FETs) [27,28], wearable gas sensors [29,30,31], and photodetectors [32,33].

Embedding 2D-layered materials in textiles could provide electronic functionality while minimizing weight and conforming to any shape, owing to their lightness, flexibility, and stretchability. These characteristics are highly desirable for developing wearable environmental control and biomedical sensors [34,35,36]. Recently, Chen et al. [37] investigated the selective gas sensing property of flexible sensors for volatile organic compounds realized from the MoS_2_ and MoS_2_-Au deposited on polyethylene terephthalate samples. To enhance the flexibility of MoS_2_-based chemical sensors on the same textiles, Kim et al. [38] combined single-walled carbon nanotubes with 2D MoS_2_ nanosheets. The obtained sensor exhibited excellent mechanical properties thanks to the carbon nanotubes reducing the cracks in the MoS_2_ layer during repetitive bending tests. Xie et al. [39] fabricated a multifunctional cellulose fabric based on MoSe_2_@MXene heterostructures exhibiting excellent photothermal performance (up to 130 °C upon irradiation for 25 s with a light intensity of 400 mW cm^−2^). Moreover, the wearable device revealed outstanding electromagnetic interference shielding effectiveness and excellent antibacterial performance [21].

Sehrawat et al. [40] investigated the potential use of a heterostructure based on WS_2_ quantum dots combined with reduced graphene oxide (RGO) to develop a novel wearable photodetector on a cotton sample. The proposed WS_2_-QDs/RGO-based photodetector showed a photoresponsivity mechanism explained in terms of charge transfer due to a suitable band alignment over the WS_2_-QDs/RGO. The same authors reported the realization of a WS_2_-QDs/RGO hybrid temperature cotton sensor that performs instant measurements in a wide temperature range (77–398 K) [22].

Despite these attempts to produce wearable sensors, to the best of our knowledge, no reports have been published on developing electrically conductive fabrics for e-textiles using 2D homostructures based on single WSe_2_.

This article presents a novel method for producing a well-distributed and homogeneous WSe_2_-based coating on textiles for wearable devices that could potentially be used as environmental moisture and temperature sensors. The process involves using an amino-functionalized silane and an acrylic thickener, yielding a coating that can be cured at a temperature lower than 170 °C. The coating is applied using an industrially scalable knife-over-roll technique to prevent any reduction in the electroconductive capacity of the dispersed WSe_2_ nanosheets within the weave structure of the textile.

## 2. Materials and Methods

### 2.1. Materials

The 2H variant of tungsten di-selenide (2H-WSe_2_) was synthesized using elemental precursors, W foil, and Se powder in an ambient pressure CVD under inert atmospheres. A 100% plain-weave cotton (scoured and bleached, with mass per unit area 331 g/m^2^, Mascioni S.p.A, Cuvio, Italy) fabric was utilized as a textile substrate for developing flexible electroconductive materials. Cotton fabrics were washed at 40 °C for 20 min using a non-ionic detergent (pH = 7) to remove undesirable contaminants and then conditioned in a climatic chamber under atmospheric pressure at 20 ± 1 °C and 65 ± 2% RH, as a standard procedure for textile materials before all experiments.

Anhydrous ethanol (ACS Reagent, Carlo Erba, Italy) and pure isopropanol (Carlo Erba, Italy) were used as solvents for developing WSe_2_ mixtures. Further, for realizing 2H-WSe_2_-containing pastes for coating the cotton fabrics, acrylic thickener with the commercial name G.E.L. (generously gifted by F.T.R. S.R.L., Albano Sant’Alessandro (BG), Italy) was used in varying amounts, both with and without N-[3-(triethoxysilyl)propyl]ethylenediamine) (EDAES) (commercial name Geniosil^®^ GF 94, purchased from Wacker Chemie AG, Stuttgart, Germany).

### 2.2. Synthetic Procedure for the Preparation of 2H-WSe_2_-Based Pastes

The exfoliated 2H-WSe_2_ powder, obtained following the exfoliation procedure as described in Supplementary Material (Par. 1.1.), was involved in a simple synthetic process for preparing three pastes, thus obtaining samples coded 2H-WSe_2__A, 2H-WSe_2__B, and 2H-WSe_2__C. To prepare samples 2H-WSe_2__A and 2H-WSe_2__B, the above-mentioned powder was dispersed in ethanol (1.5 mL) under vigorous stirring for 15 min, followed by ultrasonication treatment for 1 h at room temperature. Then, double-distilled water (3.5 mL) was added to the 2H-WSe_2_ alcoholic mixture, followed by a specific amount of acrylic thickener. During the preparation of sample 2H-WSe_2__C, EDAES was added before ethanol, and the mixture was stirred and sonicated as previously described. Then, the mixture was stirred for 2 h at room temperature to enable the hydrolysis of alkoxide groups in EDAES. Finally, the acrylic thickener was added to obtain a spreadable paste. The amount of each chemical used to prepare the three samples is reported below in Table 1.

### 2.3. Application of 2H-WSe_2_-Based Pastes onto Cotton Samples

The three 2H-WSe_2_-based pastes were deposited separately on one side of the cotton textiles using the knife-over-roll technique. Based on preliminary laboratory tests, a multilayered approach was utilized to ensure electroconductivity by depositing five 2H-WSe_2_-coating layers on an area of 7.0 cm × 1.5 cm of cotton fabrics. Textiles were dried after each layer application at 70 °C for 5 min. The textile samples (CO_A, CO_B, and CO_C) were finally cured at 120 °C for 2 min. For coating characterizations, the obtained pastes were spread on glass slides, dried, and cured, thus avoiding the interference of cotton fibers with the morphology and chemical composition of 2H-WSe_2_-based coatings.

### 2.4. Characterizations

X-ray diffraction (XRD) measurements were conducted on the bulk powder extracted from the CVD-grown WSe_2_ with a Bruker D8 advanced XRD machine (Bruker, Berlin, Germany) using a copper Kα parallel beam source, with an angle range of 2θ from 10° to 70°. The crystallinity and purity of the material were analyzed with the help of X’Pert HighScore Plus software (version 4.5). The XRD was performed as a Bragg measurement.

High-Resolution Scanning Electron Microscopy (HRSEM) measurements were performed on pristine WSe_2_ and WSe_2_-based slurries and coated textiles using Magellan 400FEI (FEI Company, Hillsboro, OR, USA). An Oxford EDS detector (with Aztec software version 2.1) with a field emission gun (Schottky field emitter) with an energy range of 500 eV–30 kV was used for the measurements under a high vacuum. Two detectors, an Everhart-Thornley detector (ETD) and a through-the-lens detector (TLD) for ultra-high-resolution SEM imaging, were used.

The surface features and heights of the WSe_2_-based slurries were investigated using a Bio Fast Scan Atomic Force Microscope (AFM) Bruker AXS (Bruker, Inc., Santa Barbara, CA, USA) under environmental conditions in an acoustic hood to minimize vibrational noise. AFM images were taken using PeakForce quantitative nano-mechanical mapping (QNM) mode with a FastScan-C silicon probe (spring constant of 0.45 N/m) and processed with a Gwyddion software (version 2.63) by directly applying the “flatting” and “planefit” functions.

High-Resolution Transmission Electron Microscopy (HRTEM) analysis of the exfoliated WSe_2_ sample and WSe_2_-based slurries was conducted in a JEM 2100 JEOL (operating at 200 keV) instrument (JEOL Ltd., Tokyo, Japan) at room temperature. Both samples were dispersed and drop cast on a Cu 300 mesh lacey carbon grid. Bright-field imaging was used for the HRTEM measurements using a single tilt holder. The HRTEM images were analyzed using Gatan Digital Micrograph software (version 3.53.4137.0 and processed further for quality and clarity in Image-J, version 1.4.3.67).

X-ray Photoelectron Spectroscopy (XPS) measurements on WSe_2_-based slurry samples were performed using the Thermo Scientific Nexsa spectrometer (East Grinstead, UK) with a mono-chromated, micro-focused, low-power Al K_a_ X-ray source (photon energy 1486.6 eV). Samples were irradiated with a soft X-ray source (~1.5 keV) under ultra-high vacuum at a base pressure of 5 × 10^−10^ torr (no higher than 3 × 10^−9^ torr). XPSPEAK software (version 4.1) was used to analyze the data. Survey and high-resolution spectra were acquired at pass energies of 200 eV and 50 eV, respectively. The source power was set at 72 W. Unless otherwise specified, the binding energies were recalibrated by setting the CC/CH component of the C 1 s peak at 285 eV. Qualitative surface chemical analysis was performed using high-resolution core-level spectra after eliminating a nonlinear Smart background.

The chemical composition of the 2H-WSe_2_-conductive pastes and the presence of these coatings on cotton samples were characterized by Fourier Transform Infrared (FTIR) Spectroscopy using a Thermo Nicolet Avatar 370 spectrophotometer (Madison, WI, USA) equipped with an attenuated total reflection (ATR) accessory and a diamond crystal as the internal reflectance element. The spectra were recorded in transmittance and absorbance (for glass slides and textile samples analysis, respectively) ranging between 4000 cm^−1^ and 550 cm^−1^, performing 32 scans with a resolution of 4 cm^−1^. ATR-FTIR cotton fabric curves were normalized at 1335 cm^−1^ (O-H in-plane bending) [41].

In this study, the humidity- and temperature-sensing properties of WSe_2_-containing fabrics were investigated through electrical resistance measurements. This type of characterization was selected since the sensor needs to be integrated into wearable ecosystems for environmental parameter monitoring. In such applications, which operate in low-power and low-frequency scenarios, the DC voltage and current are known for a nominal point, and variations in environmental parameters are inferred from changes in the DC current over time. Furthermore, the transients between different environmental conditions in real-world scenarios occur relatively slowly, typically in the order of minutes. Following this reasoning, the reactance behavior of the sensor can be neglected as a first approximation, even though it is of great interest from an experimental perspective. However, the study of reactance is beyond the scope of the current investigation.

To investigate the sensor response of WSe_2_-based textile sensors, electrical resistance measurements were performed on treated fabrics at different humidity levels and temperatures under controlled temperature and humidity conditions, respectively. The test setup involved fixing a sample on plexiglass support using two metal electrodes inside an environmental test chamber (Angelantoni ACS DY110, Angelantoni Test Technologies, Massa Martana (PG), Italy), which was used for the generation, setting, and control of both humidity and temperature values during the whole experiment. The electrodes were connected to an external source measurement unit (SMU) (Agilent B2961A Low Noise Power Source, Agilent Scientific Instrumentation, Santa Clara, CA, USA) to bias the sample at a fixed voltage of 20 V.

To investigate the environmental humidity sensing of WSe_2_ nanosheet-based fabrics, each sample was exposed to different RH% levels. To minimize temperature fluctuations caused by changes in environmental humidity, the temperature inside the climatic chamber was maintained at 25 °C ± 0.06 °C. The humidity inside the climatic chamber was made to rise in the 30–90% ± 0.40% range (by setting a 10% RH ramp in the range of 30–70% ± 0.57% RH, and a 5% RH ramp between 70% ± 0.68% and 90% ± 0.35% RH). Then, the RH% of the chamber was returned to 30% by setting the same RH% ramps already described for the experiment between 30 and 70% RH, and the entire cycle was repeated four times to assess the sensing repeatability of each sample. An initial stabilization period of three hours was implemented, followed by intervals of stabilization for each RH% level set at an average of 30 min.

Following a similar procedure, a second experiment was performed on identical textile fabrics by investigating the electrical resistance changes as a function of different temperatures. To minimize humidity fluctuations resulting from temperature variations, the relative humidity inside the climatic chamber was maintained at 50% ± 0.49%. The temperature inside the climatic chamber was swept between 20 °C ± 0.03 °C and 30 °C ± 0.03 °C (with increments of 5 °C), then up to 60 °C ± 0.05 °C (with increments of 10 °C). Then, the temperature was returned to 20 °C ± 0.04 °C by setting the same temperature increments already described for the experiment between 20 and 60 °C, and the entire cycle was repeated four times. After an initial settling time of five hours, the temperature was modified every 2.5 h to obtain the appropriate values described earlier, thus leaving the sample to stabilize.

Due to the small thickness of the 2H-WSe_2_ deposited on cotton fabrics (estimated at approximately 100 μm), it was possible to refer to the surface or sheet resistance (R_s_) [42]. Accordingly, the conductive properties of the 2H-WSe_2_-treated fabrics were assessed by referring to the resistance over a fixed aspect ratio area [43]; R_s_ was calculated as per Equation (1).

(1) $$R_{S}=R\frac{W}{L}\left[\frac{\Omega }{sq}\right]$$

As an essential parameter for sensor performance, the sensitivity of the sensor film was defined as the ratio of the resistance at a set humidity level (R_RH_) to the resistance measured at the lowest set humidity (R_RL_), following Equation (2) [44]:

(2) $$S\left(\%\right)=\frac{∆R}{R_{RL}}=\frac{\left(R_{RH}-R_{RL}\right)}{R_{RL}}\times 100\%$$

To investigate the underlying mechanism of the functioning of the temperature sensor of the developed 2D-based textiles, crucial parameters—such as the temperature coefficient of resistance (TCR, %) and thermal hysteresis (H_th_)—were calculated according to the following equations [22]:

(3) $$TCR\left(\%\right)=\frac{1}{R_{0}}\frac{∆R}{∆T}\times 100\%$$

(4) $$H_{th}=\frac{\left(R_{0}-R_{f}\right)}{R_{0}}\times 100\%$$

In Equations (3) and (4), R_0_ and R_f_ are the resistance of samples corresponding to the initial and the targeted temperatures, respectively; ΔR and ΔT are the resistance and temperature changes, respectively. Both parameters were calculated directly by monitoring the textile sensor response to the increase in temperature from the lowest temperature to a target one, up to 60 °C.

The response times of the WSe_2_-doped textile sensors were evaluated as the time difference between the instants when the resistance value reached 10% and 90% of its final stabilized value.

The influence of WSe_2_ coatings on mechanical properties and comfortability was evaluated through the bending length (LB) index, which reveals a decreased softness with increased stiffness [9]. The bending stiffness of the textile samples was measured according to DIN 53362:2024-11[45] in terms of sample length to achieve the bending angle of 41.5°. The material was tested in two directions and the mean values were calculated from five repetitions with a standard deviation lower than ±5%.

## 3. Results and Discussion

Chemical and morphological characterization findings referring to developed WSe_2_ nanosheet composites are summarized in this section. The details of the WSe_2_ synthesis [46,47,48,49], as well as related phase purity images, are reported in the Supplementary Material (Figures S1–S3).

### 3.1. ATR-FTIR Spectroscopic Analysis of 2H-WSe_2_-Based Slurries

**Figure 1:**
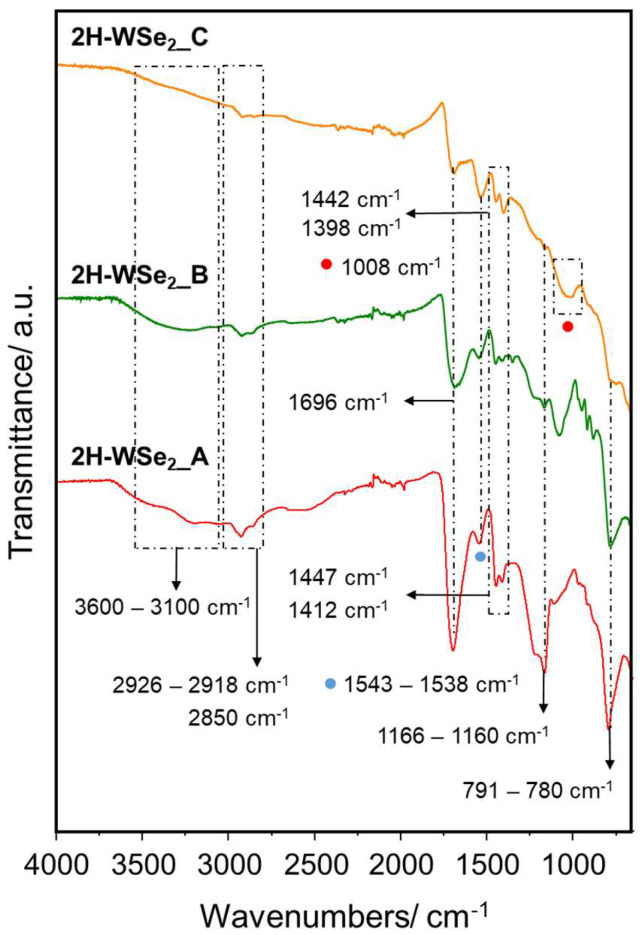
ATR-FTIR spectra of 2H-WSe_2__A, 2H-WSe_2__B, and 2H-WSe_2__C coatings on glass slides. The colored dots highlight the absorption bands and the assigned wavenumbers.

The chemical composition of the prepared 2H-WSe_2_ slurries was preliminarily studied as a solid residue on glass slides, prepared following the same procedure described in Section 2.3 for textiles, thus avoiding the interference of cellulose absorbance bands. Figure 1 depicts the infrared spectra of 2H-WSe_2__A, 2H-WSe_2__B, and 2H-WSe_2__C slurries. The characteristic peaks of WSe_2_ nanosheets at approximately 590 cm^−1^ (W-Se vibrations) were not observed due to the instrument’s detection limit [50]. However, in all spectra of 2D-based coatings, the prominent characteristic bands relative to the acrylic thickener were observable. The broad band at 3600 cm^−1^–3100 cm^−1^, peaks at 2918 cm^−1^–2926 cm^−1^, and 2850 cm^−1^ were assigned to O–H and C–H stretching, respectively [51,52]. The carboxyl functionality of the acrylic thickener was attributed to the peak at 1696 cm^−1^ in WSe_2__A that assumes a general characteristic and a shift to 1685 cm^−1^ in the coating 2H-WSe_2__B due to enhanced interaction between WSe_2_ (doubled concentration to 2H-WSe_2__A) and the acrylic thickener. The intensity of the carboxylic group in 2H-WSe_2__C was reduced to 1683 cm^−1^ because of changes in the chemical environment for the presence of EDAES. Similar shifts were observed for bands at 1447 cm^−1^ and 1412 cm^−1^ (Figure 1, red curve) relative to CH_2_ deformation and C–O stretching coupled with O–H in-plane bending, which moved to 1442 cm^−1^ and 1398 cm^−1^ when EDAES was added to the polymeric matrix [51]. Out-of-plane bending vibrations of C–H bonds were observed in the 2H-WSe_2_ coatings at 1166 cm^−1^–1160 cm^−1^ [53]. Moreover, the peaks at 1543 cm^−1^–1538 cm^−1^ and 791 cm^−1^–780 cm^−1^ are assigned to the stretching vibration of C = C [54]. At approximately 1008 cm^−1^, a broad band in the 2H-WSe_2__C coating confirmed the presence of the silane matrix [55] and the formation of a self-assembled silica network monolayer onto layered 2H-WSe_2_, while other characteristic peaks of Si-O-Si (at approximately 810 cm^−1^) [9] were not observed since highly intense peaks of acrylic thickener overlapped them.

### 3.2. Understanding the Homogeneity of 2H-WSe_2_-Based Coatings

**Figure 2:**
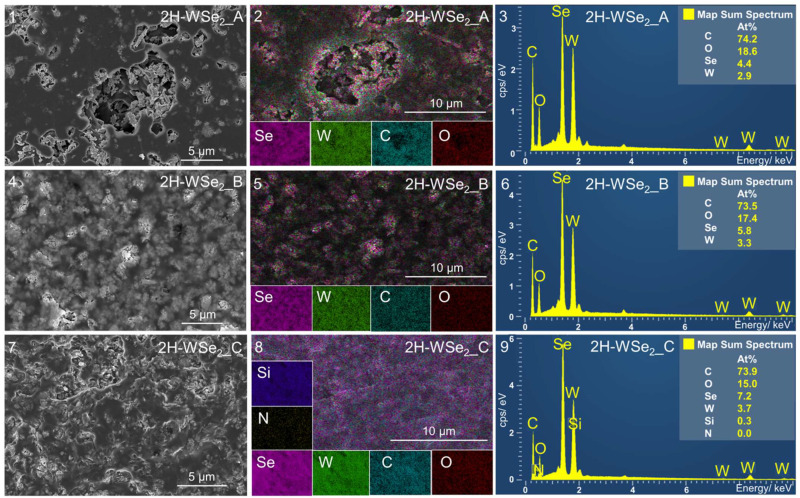
HRSEM images of 2H-WSe_2_ slurries A (**1**), B (**4**), and C (**7**), corresponding HRSEM containing overlapped EDS images of C, Se, W, O ((**2**,**5**,**8**) for samples A, B, and C, respectively) and EDS spectra ((**3**,**6**,**9**) for samples A, B, and C, respectively).

The quality of the slurries deposited on the glass slides containing different ratios of acrylic thickener and WSe_2_ was assessed. The coated glass slides were prepared following the same procedure described in Section 2.3 for textiles. The HRSEM image at a lower magnification of slurry A is depicted in Figure 2(1), while the overlapping images from the area-mapping scan are depicted in Figure 2(2). The presence of C, Se, W, and O is confirmed from the individual area maps as per the insets in Figure 2(2). As mentioned, the qualitative energy dispersive spectroscopy (EDS) map from Figure 2(3) indicates the elements’ presence. Similarly, slurries B and C in Figure 2(4,7) are also analyzed. The overlapping images from the area-mapping scan are depicted in Figure 2(5) and Figure 2(8), respectively. The presence of C, Se, W, and O in slurry B is confirmed from the individual area maps as per the insets in Figure 2(5), while the presence of Si and N in slurry C is also depicted (Figure 2(8)). The EDS map from Figure 2(6) and Figure 2(9) also confirms the elements in slurries B and C, respectively.

The SEM images and EDS measurements reveal the nature of WSe_2_ dispersions in the prepared slurries by mixing them with the acrylic polymer. The surface SEM images indicate a few areas that contain WSe_2_ nanosheets.

To support and identify the appropriate incorporation of the WSe_2_ nanosheets into the polymer matrices, TEM analysis of the WSe_2_-based polymer slurries A, B, and C was performed. A close examination of different positions of the Cu TEM grid revealed how the WSe_2_ nanosheets are embedded into the polymer matrices. A closer inspection of HRTEM images of slurry A (Figure S4(2,4)) indicates the (002) fringes as evident from the Fast Fourier Transform (FFT) images in the insets. The selected area diffraction (SAED) from position 2 also confirms the existence of the (100), (103), and (008) planes (Figure S4(5)). Moreover, the EDS measurements from position 2 align with those previously detected in the HRSEM (Figure S4(6)). Similarly, in Figure S4(7,9), the presence of WSe_2_ in slurry B is confirmed as per the corresponding HRTEM images in Figure S4(8,10), in which the FFT displays a clear honeycomb. The presence of (112), (100), and (008) planes are also confirmed in the SAED in Figure S4(11), with EDS confirmation in Figure S4(12). Figure S4(13–16) also depict the acrylic polymer C mixed with WSe_2_. Additionally, the (111) Si plane was identified in the SAED as per Figure S4(17). However, due to the overlap of intensity with W in the EDS (Figure S4(18)), the Si peak cannot be distinctly resolved. As explained before, the HRTEM measurements of slurries are also in complete agreement with the AFM measurements.

The AFM measurements (Figure S5) revealed the height of the WSe_2_ nanosheets incorporated into the acrylic polymer that are summarized below:

Slurry A: Figure S5(1) Height Profile: 4.5 ± 0.3 nm, and Figure S5(2) Height Profile: 2.6 ± 0.3 nm;

Slurry B: Figure S5(3) Height Profile: 3.1 ± 0.3 nm, and Figure S5(4) Height Profile: 6.3 ± 0.3 nm;

Slurry C: Figure S5(5) Height Profile: 4.4 ± 0.3 nm, and Figure S5(6) Height Profile: 4.5 ± 0.3 nm.

XPS analysis, detailed in the Supplementary Material, evidenced surface oxidation and the nature of chemical bonding between the WSe_2_ nanosheets and the polymers. The spectra (Figure S6) show W 4f, Se 3d, C 1s, O 1s, Si 1s, and N 1s levels, indicating interaction and slight oxidation in various slurries.

### 3.3. Chemical Characterization of 2H-WSe_2_-Coated Textiles

**Figure 3:**
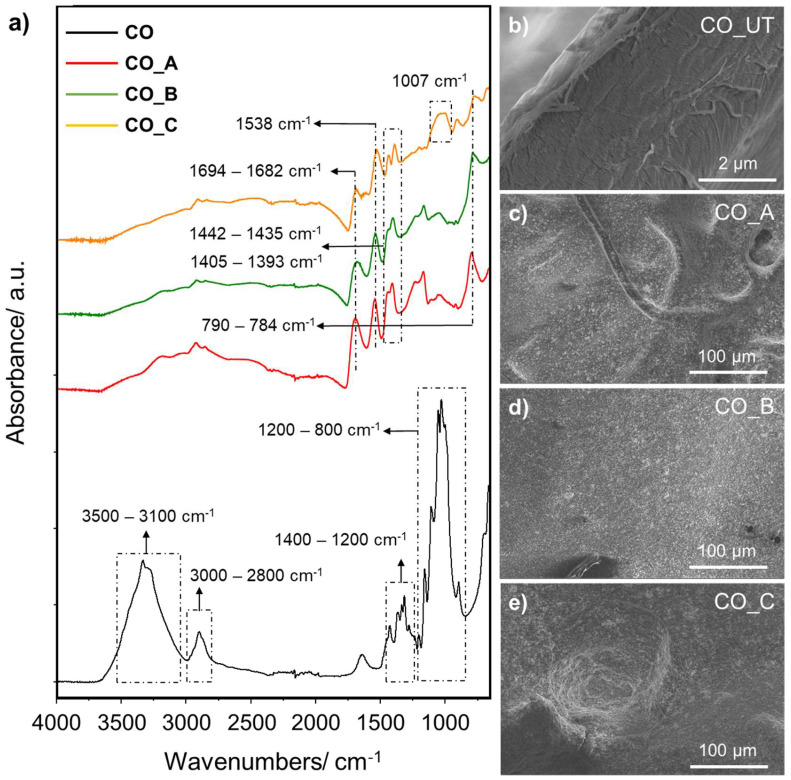
ATR-FTIR spectra of untreated and treated cotton samples with 2H-WSe_2_ nanosheet-based coating A, B, and C (**a**); SEM images of CO_UT (**b**), CO_A (**c**), CO_B (**d**), and CO_C (**e**) fabrics.

According to ATR-FTIR spectroscopy, compared to the reference cotton (CO_UT) (Figure 3 on the left, black curve), treated samples are characterized by absorption bands, which distinguish the chemical structure of the applied coating, as already discussed in Figure 1. These bands covered the typical absorption bands of cellulose moiety assigned to O–H stretching (3500 cm^−1^–3100 cm^−1^), C–H stretching (3000 cm^−1^–2800 cm^−1^), C–H wagging, and deformation mode (1400 cm^−1^–1200 cm^−1^), asymmetric stretching of C–O–C, in-plane ring, and C–O stretching (1200 cm^−1^–800 cm^−1^) [56,57], thus confirming the homogeneous distribution of WSe_2_ coating onto cotton surfaces.

In contrast, an estimation of the homogeneity of the materials is also qualitatively assessed using HRSEM. Figure 3b reveals a typical morphology of untreated cotton fabric that appears characterized by a smooth and clear surface structure, with randomly natural convolutions or twists along some fibers. The surface characteristics of pristine cotton significantly influence coating adhesion, since convolutions may act as localized anchoring points. To a greater extent, the hydroxyl groups on cellulose fibers enable chemical bonding with reactive groups in coatings, such as silanols in sol-gel or functional groups in the acrylic thickener, ensuring adhesion between the coating and the cellulose-based substrate. Indeed, the coatings on the textiles appear uniform throughout since the characteristic smooth morphology of cotton fibers is no longer observable (Figure 3c, Figure 3d, and Figure 3e, relative to CO_A, CO_B, and CO_C, respectively). Accordingly, the coatings have a high degree of coverage and are rough with a porous structure that helps water molecules interact with the sensitive WSe_2_-layered material.

## 4. Humidity and Temperature Sensing of the 2D-WSe_2_-Coated Fabrics

In this paragraph, the sensing performance of the developed WSe_2_ nanosheet-based textiles in detecting environmental temperature or humidity is investigated and thoroughly discussed.

As reported in the literature, the deposition of conductive materials on textile surfaces significantly increased the surface conduction properties of the treated fabrics by revealing lower electrical resistance values than the untreated fabric surface [9,58,59]. Indeed, 2H-WSe_2_-treated cotton samples characterized before experiments showed, at room humidity and temperature (50 ± 0.44 RH% and 25 ± 0.004 °C), average Rs values of 1.57 × 10^9^ ± 4.83 × 10^7^ Ω sq^−1^, 2.87 × 10^8^ ± 1.16 × 10^7^ Ω sq^−1^, and 1.14 × 10^9^ ± 2.53 × 10^7^ Ω sq^−1^ for samples A, B, and C, respectively, when measured in the air. Moreover, the deposition of a higher concentration of 2H-WSe_2_ on cotton lowers Rs values by approximately one order of magnitude (sample B versus sample A).

According to the percolation theory [60], this phenomenon is also affected by structural parameters (crystal structure and related anisotropy) as well as network connectivity (among the flakes). Indeed, increased conductivity is expected due to the low activation energy and short hopping distance caused by a large density of Se vacancies [61].

### 4.1. Humidity-Sensing Performance of 2D-Doped Cotton Fabrics

**Figure 4:**
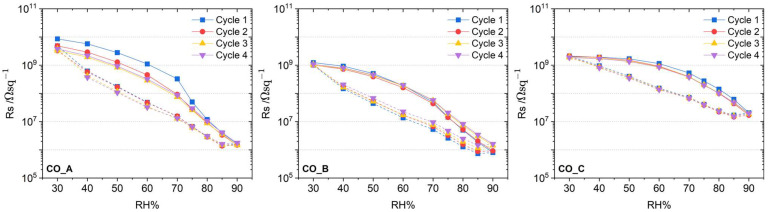
Surface resistance versus RH% at room temperature for CO_A, CO_B, and CO_C during four exposure cycles: from 30% to 90% RH (solid lines) and from 90% to 30% RH (dashed lines). The standard deviation was between ±7% and ±14% for lower and higher RH%, respectively.

The response of the developed 2D-based conductive fabrics to humidity at room temperature is depicted in Figure 4.

Graphs in Figure 4 exhibit an inverse proportionality of Rs values to RH% levels. Considering the maximum and minimum Rs values obtained in this experiment (Table S1), it can be stated that the Rs of treated samples range over three orders of magnitude in samples A and B and over two orders of magnitude in sample C, thereby further indicating a significant cycle hysteresis.

During the first humidity exposure cycle (Figure 4, blue curve), the CO_A cotton samples provided the highest Rs values when the RH% was between 30% and 75%; moreover, the almost overlapping between the Rs values during the subsequent cycles (Figure 4, CO_A, red, yellow, and violet curves) indicate a repeatable response to humidity. When the chamber moisture level was returned from 90% RH to 30% RH (Figure 4, CO_A, dashed lines), the lowest Rs values were measured for each exposure cycle but for the same magnitude. In particular, the highest conduction properties of sample A were observed for the third and fourth humidity exposure cycles, both from 30% to 90% RH and from 90% to 30% RH (Figure 4, yellow and violet solid/dashed curves, respectively).

Sample CO_B revealed a similar humidity response trend to sample A but with slight differences, particularly from 90% to 30% RH. Until 75% RH, sample B provided similar Rs values regardless of the number of humidity exposure cycles. However, when the RH% was increased from 75% to 90%, the number of moisture exposure cycles affected the Rs of the sample: the higher the exposure cycles, the higher the Rs values and the lower the electrical conductivity of the samples. This trend was also observed from 90% to 50% RH. However, compared to sample A, sample B proved to be slightly more conductive, between 30% and 80% RH, since the Rs values measured were one order of magnitude lower due to the higher WSe_2_ concentration used.

In contrast, sample C revealed a similar trend to sample A in the conduction properties, although it is less conductive (two orders of magnitude of difference) when exposed to humidity between 75% and 90% RH and between 90% and 60% RH. These lower conduction properties of sample C can be due to the presence of the sol-gel matrix acting as a spacer between WSe_2_ nanosheets in the coating, thus affecting ion conduction.

Overall, for high RH% ranges, the response time is longer when humidity is increased from 30% to 90% RH than when RH% is returned from 90% to 30% RH (Supplementary Table S2). This can be explained through the high water affinity of WSe_2_-layered material, the water entrapment between coating layers, and the hydrophilicity of cotton fabric, which significantly contribute to water uptake and, thus, influence the response time of the material. As the literature lacks studies on WSe_2_ homostructures as sensing materials and only reports on TMD heterostructures [13] based on junctions between different 2D materials, the response times measured for all samples demonstrate an acceptable humidity response and reversibility [13].

Following literature data [44], the observed experimental findings can be explained by referring to the doping behavior of the adsorbed moisture on WSe_2_ nanosheets. In this regard, the humidity-sensing mechanism can be explained by proton conduction, according to the Grotthuss mechanism [62], apart from the electron/hole transfer [62,63]. According to this mechanism, protons (H^+^) are tunneled between water molecules by hydrogen bonding [63]. At low RH% levels, water molecules are chemisorbed on the WSe_2_ nanosheet surface, thereby dissociating in hydroxyl (OH^−^) and proton (H^+^) ions, as shown by Equation (5) [62].

Furthermore, at this stage, the first physisorption phenomena begin to occur.

(5) $$H_{2}O↔H^{+}+{OH}^{-}$$

However, at these low RH% values, the primary sensing mechanism is based on electron transfer from water to p-type WSe_2_ since the first physisorbed water layer is not continuous and protons (also from the chemisorbed layer) cannot move freely and support conduction [19].

Increasing the RH% levels, additional water molecules are physisorbed through hydrogen bonds on the chemisorbed layer [19], further dissociating in hydronium (H_3_O^+^) and OH^−^ ions (Equation (6)).

(6) $$2H_{2}O↔H_{3}O^{+}+{OH}^{-}$$

**Scheme 1:**
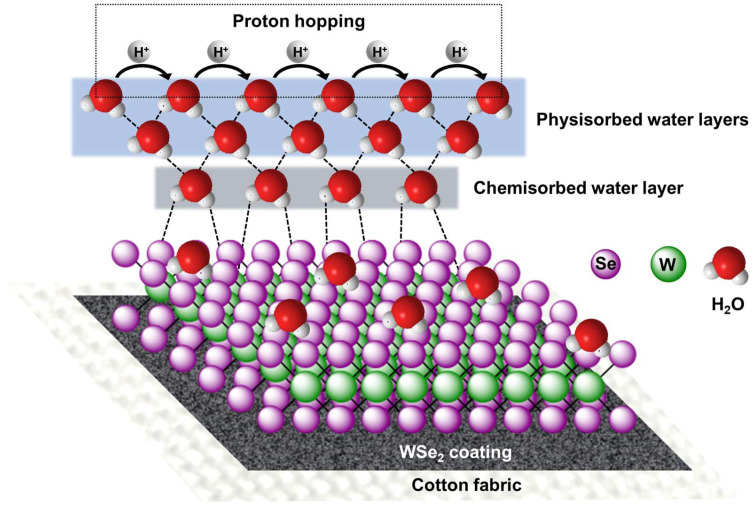
Schematic representation of the humidity-sensing mechanism of 2H-WSe_2_ nanosheet-based cotton fabrics according to the Grotthuss mechanism.

Due to multilayered physisorption, a large number of H_3_O^+^ are generated, causing a sharp rise in the sensing response produced by proton conduction through hopping following the Grotthuss chain reaction (Equation (7)) [19,64,65], which can take place in both water layers (Scheme 1).

(7) $$H_{2}O+H_{3}O^{+}\to H_{3}O^{+}+H_{2}O$$

Consistent with this mechanism, the surface resistance of the developed WSe_2_-based textile sensors decreases with increasing humidity due to the adsorption of water molecules, which establish conduction pathways between the 2D nanosheets. In contrast, for CNT-based textile sensors, the predominant humidity-sensing mechanism is coating swelling, which leads to an increase in R_s_ as humidity rises [58,59]. During water adsorption, nanoscale gaps form between the WSe_2_ sheets, facilitating further water penetration between layers [16,44]. Consequently, the adsorbed water molecules induce a shift in the Fermi energy, transitioning the semiconducting nature of WSe_2_ toward the conduction band [16,44].

However, the multilayered coating structure and its chemical composition can influence the humidity-sensing mechanism of the developed fabrics by enhancing water diffusion through the film deposited on the cotton surface. The hydrophilic nature of the cotton further increases water adsorption by the coating [66]. As predicted by the physisorption phenomena, the higher the water adsorbed by the coating, the higher the ion mobility; this increases the humidity level [67].

When humidity levels are returned (from 90% to 30% RH), an expected increase in the resistance values was observed for all samples during the four cycles caused by the evaporation of water molecules from the WSe_2_-doped films and leading to a reduction in proton conduction. Here, the release of water from the interlayer on the sensor was facilitated by the multilayered coating structure that governs the dynamic equilibrium between internal and external humidity levels [68].

**Figure 5:**
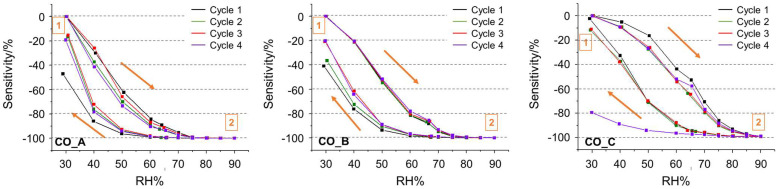
Sensitivity of 2H-WSe_2_ nanosheet-based cotton fabrics (CO_A, CO_B, and CO_C) at set RH% calculated following Equation (2) for the four humidity exposure cycles.

Therefore, the number of water molecules adsorbed/desorbed on/from 2D-doped film significantly influences the sensitivity of the developed composite sensor. As evident from Figure 5, the developed sensors exhibit negative sensitivity, equal to or close to 100% for high moisture levels; in general, the lower the sensitivity values, the higher the moisture level. These negative values can be ascribed to the adsorption of water vapor molecules in a saturated environment, which shifts the Fermi energy toward the conduction band [44]. It is crucial to note that when humidity is returned from 90% to 30% RH (Figure 5, arrow from point “2” to “1”), lower sensitivity values than that of the corresponding RH%, when humidity is increased from 30% to 90% RH (Figure 5, arrow from point “1” to “2”) were calculated. This finding indicates excellent repeatability in recovery behavior, particularly for samples A and B. The influence of the silane network in the 2H-WSe_2_-based coating on the conductive properties of the composites was again confirmed in the recovery cycles (Figure 5, CO_C).

Composites with high concentrations of WSe_2_ nanosheets show better humidity-sensing performance. The inorganic 2H-WSe_2_-layered material doped in polymeric textile coatings has potential application in humidity detection.

### 4.2. Temperature-Sensing Performance of 2D-Doped Cotton Fabrics

**Figure 6:**
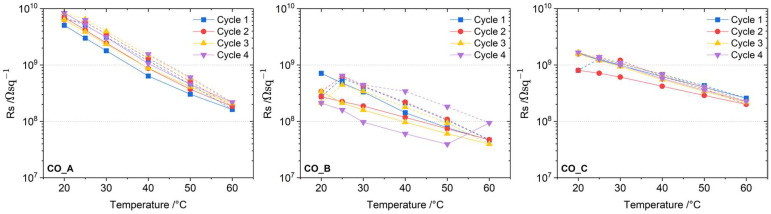
Surface resistance versus temperature for CO_A, CO_B, and CO_C during four exposure cycles: from 20 °C to 60 °C (solid lines) and from 60 °C to 20 °C (dashed lines). The standard deviation was between ~1% and ~6% for higher and lower temperatures, respectively.

The developed 2H-WSe_2_ nanosheet-based humidity sensors were further investigated as potential resistance temperature detectors, as described in the experimental section, and the sensor responses are reported in Figure 6.

Graphs in Figure 6 highlight an inverse proportionality of R_s_ values to temperature (particularly evident for sample A) as well as R_s_ values ranging from almost two, almost one, and precisely one order of magnitude in samples A, B, and C, respectively (Supplementary Table S3).

CO_A yielded the lowest R_s_ values in the range 20–60 °C (Figure 6, CO_A, blue solid lines), while when the chamber temperature was returned from 60 °C to 20 °C (Figure 6, CO_A, dashed curves), R_s_ values were slightly higher, thereby confirming the repeatability of temperature-electrical conduction properties of 2D-based textiles.

Unlike the humidity-sensing experiments, where the CO_B sample demonstrated good recovery capability by returning to its initial R_s_ values during the humidity recovery cycles, the same sample exhibited a lower recovery capability in the temperature-sensing experiments. As shown in Figure 6, CO_B displayed an increasing resistance deviation with a higher number of temperature exposure cycles, highlighting its lower recovery capability compared to CO_A. Nevertheless, even in this experiment, CO_B maintained higher conductivity than CO_A. As explained in the previous paragraph, the highest conductivity in sample B could be ascribed to the higher WSe_2_ concentration and temperature-dependent conductivity transport mechanism [61].

Sample CO_C showed close conduction properties both in the temperature increase and recovery cycles (20 °C to 60 °C and 60 °C to 20 °C, respectively), with a conductive curve trend closer to that shown by sample CO_A than CO_B. Moreover, despite the high concentration of WSe_2_ used in coating C, the presence of the sol-gel matrix led to higher R_s_ values than those for sample CO_B.

As observed above, the resistance of the realized temperature sensors decreases when the temperature increases, and an overall recovery of the R_s_ was observed for tested samples when the temperature was returned to 20 °C (except for sample CO_B).

As reported earlier [69,70], the increase in conductivity of 2D-based temperature sensors under temperature variation is due to higher charge carrier concentration. Notably, the electrical resistance of the 2D-based cotton fabrics revealed a quadratic temperature dependence in the range of 20–60 °C, acting as resistance temperature detectors. According to [71], the conductivity rise of 2D materials is strictly related to an increase in the electron hopping transport.

Temperature-dependent conductivity based on thermal activation occurs via nearest neighbor hopping (NNH) [72,73], which is usually expected at elevated temperatures as lower temperatures increase hopping distance. Furthermore, high temperatures ensure excellent charge transportation and high charge density [74], enhancing electrical conduction by generating more carriers by WSe_2_ [75]. However, temperature-dependent electron mobility can be affected by several other scattering mechanisms (e.g., surfaces, edges, defects, and phonons) [76,77,78] that are challenging to identify in micro-scale devices.

**Figure 7:**
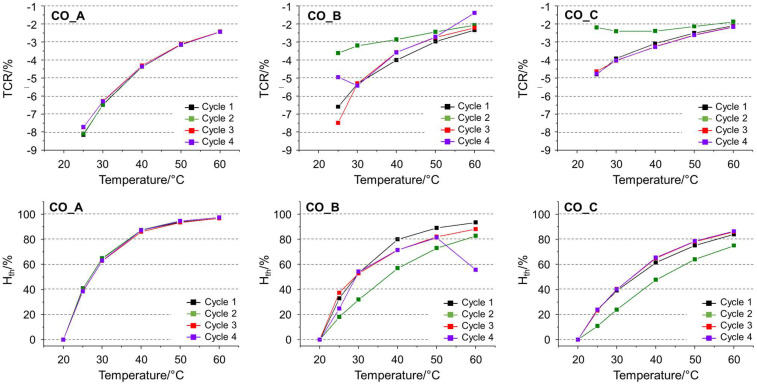
TCR (%) and H_th_ (%) plots versus temperature for 2H-WSe_2_ nanosheet-based cotton fabrics (CO_A, CO_B, and CO_C) for the four exposure cycles (from 20 °C to 60 °C), calculated following Equations (3) and (4), respectively.

In Figure 7, TCR and H_th_ plots as a function of temperature for 2H-WSe_2_ nanosheet-based cotton fabrics are reported to demonstrate the sensitivity and efficiency of each 2D-based textile sensor. According to the literature [79], an efficient sensor should provide high TCR and low H_th_.

TCR varied from −8.15% to −2.41%, moving from 20 °C to 60 °C for the first exposure cycle of sample CO_A. The TCR% at 20 °C reduced from −8.16% to −7.72% after four exposure cycles, while the TCR% at 40 °C and 60 °C was almost constant. Sample CO_B revealed TCR% values lower than those of CO_A but characterized by a non-constant trend with the progress of temperature exposure cycles, thereby revealing the highest TCR value of −7.48% at 20 °C (third cycle) and the lowest value of −1.39% at 60 °C (fourth cycle). Conversely, sample CO_C provided the lowest TCR% (approximately −4.7%) measured at 20 °C by maintaining similar TCR% values of samples A and B at 60 °C. According to literature data, an increase in the TCR value corresponds to increased sensor response and, consequently, sensitivity [22]. Experimental data demonstrated good responsivity, stability, and semiconductor behavior of WSe_2_-based textiles, as confirmed by the negative TCR%, whose high values can be ascribed to the thermally excited electrons of hexagonal WSe_2_ nanosheets [80].

Beyond, H_th_ estimates the retention of heat energy, thus defining the extent of sensor efficiency concerning reversibility. Contrary to existing data [20,22], H_th_ measured for these 2D-based textile sensors (Figure 7 below) is not negligible but increases with increasing temperature until attaining values of approximately 100 for temperatures above 40 °C. In particular, the highest values were calculated for the CO_A sample at approximately 96–97% at 60 °C, while the lowest values were obtained from sample CO_B and ranged between 93.43% and 55.69% at 60 °C for cycles 1 and 4, respectively. The presence of sol-gel in the WSe_2_ coating in sample CO_C recovered the H_th_ values close to those measured for sample CO_A in the range of 75.06–86.74%.

The experimental findings demonstrated that the obtained coatings containing WSe_2_ homostructures are characterized by typical thermal hysteresis behavior, which is in contrast to that in the literature for other TMDs-based homostructures (e.g., MoSe_2_) [20] or heterostructures (e.g., WS_2_-QDs) [22], thus highlighting the potentiality of the developed coating in sensor applications.

The performances obtained in terms of humidity and temperature sensing for the developed WSe_2_ textiles are shown in Table 2, compared with other textile-based TMD sensors. Currently, the integration of TMDs into textiles for humidity and/or temperature monitoring remains an emerging area of research with limited practical examples. Most TMD-based sensors have been developed on rigid or flexible non-textile substrates. To the best of our knowledge, the following table presents TMD-based humidity and temperature textile sensors available in the scientific literature in comparison with this current study.

## 5. Stiffness Properties

Generally, applying a coating that binds individual fibers is anticipated to alter their mobility, thereby increasing the stiffness of the treated fabric. Indeed, the degree of stiffness in textiles is a critical determinant of comfort and functionality. While coatings can enhance properties such as durability, water resistance, or antimicrobial activity, they may also impact the flexibility of treated fabrics. Experimental findings indicate a slight rise in stiffness across all coated samples compared to untreated cotton. Specifically, the measured lengths of the samples were 55 mm, 57 mm, and 60 mm for the three coated variants, in contrast to 50 mm for the uncoated reference. This suggests that the coatings induce a slight stiffening effect. However, the observed increase in stiffness in the treated samples remains within acceptable limits, indicating that the coatings do not significantly compromise the fabric’s comfort. This balance between enhanced functional properties and maintained flexibility is crucial in textile applications, ensuring that the end product meets performance and user comfort standards, making them suitable for various applications.

## 6. Conclusions

The WSe_2_-based coatings developed in this study successfully established conductive networks on the insulating cellulose surface without significantly compromising fabric comfort. These WSe_2_-coated cotton textiles exhibited promising humidity- and temperature-dependent electrical resistance across various environmental conditions. Notably, all samples displayed consistently high negative temperature coefficient of resistance (TCR) values, characteristic thermal hysteresis behavior, and good responsivity and stability over multiple temperature exposure cycles.

Furthermore, this study highlights the advantages and potential applications of the inorganic 2H-WSe_2_-layered material incorporated into polymeric textile coatings for reliable humidity and temperature sensing. The integration of WSe_2_ within textile substrates offers a promising approach for developing flexible, wearable environmental sensors.

Nevertheless, further research is required to assess the long-term durability of the electroconductive coatings deposited on cotton fabrics, particularly under repeated mechanical stress and prolonged exposure to varying environmental conditions. Additionally, optimization of the sensing performance, including sensitivity, response time, and recovery behavior, will be crucial for advancing the practical application of WSe_2_-based textile sensors in real-world scenarios.

## Figures and Tables

**Table 1:** Amount of chemicals in the synthetic preparation of 2H-WSe_2_-based pastes; photos of samples.

Sample Code	2H-WSe_2_ (mg)	Acrylic Thickener (mg)	EDAES (μL)	Acrylic Thickener: 2H-WSe_2_	Sample Photo
2H-WSe_2__A	77.6	165.7	-	2.1	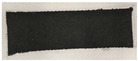
2H-WSe_2__B	195	148.2	-	0.8	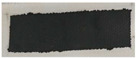
2H-WSe_2__C	197.5	136.3	33	0.7	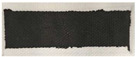

**Table 2:** Comparison of sensing properties among different humidity and temperature TMD-based textile sensors.

Type	Textile Substrate	Measurement Range and Sensing Parameters						Ref.
		%RH	°C	Sensitivity	TCR%	H_th_%	Response/Recovery	
WS_2_/Ti_3_C_2_T_x_	cotton	40–90%	-	1.61787	-	-	~20.93s/~233.87s	[81]
MoSe_2_@ MXene/CFs	cotton	-	20–160 °C	-	~−1.3 × 10^−3^	-	-	[39]
WS_2_-QD/RGO	cotton	-	298–398 K	-	–0.58	5.22	92.65s/144.16s	[22]
WSe_2_	cotton	30–90%	20–60 °C	~−100% (at high %RH)	−2.41 (at 60°)	~96 (at 60 °C)	* 10–30min (%RH cycles) * 1.8 h–2.5 h/1.5 h (temp. cycles)	This work

* Mean measured ranges for response times during humidity- and temperature-sensing tests, as reported in Table S2 and Table S4, respectively.

## Data Availability

The original contributions presented in this study are included in the article/Supplementary Material. Further inquiries can be directed to the corresponding authors.

## Supplementary Materials

The following supporting information can be downloaded at: https://www.mdpi.com/article/10.3390/polym17060752/s1, Figure S1: Schematic representation for tungsten di-selenide (2H-WSe_2_) growth on W foil (using ambient pressure CVD), deposition of a few layers of 2H-WSe_2_ on the surface of the W foil, and furnace setup; Figure S2: (1) High-Resolution Scanning Electron Microscopy (HRSEM) image of bulk tungsten di-selenide (2H-WSe_2_); (2) Atomic Force Microscopy (AFM) measurement of exfoliated pristine 2H-WSe_2_ nanosheets; (3) High-Resolution Transmission Electron Microscopy (HRTEM) image of exfoliated pristine 2H-WSe_2_ nanosheets on Cu Grid; (4) High-Resolution Transmission Electron Microscopy (HRTEM) image of exfoliated pristine 2H-WSe_2_; (5) Selected Area Diffraction (SAED) pattern from WSe_2_ nanosheets; Figure S3: X-Ray Diffraction (XRD) pattern of bulk tungsten di-selenide (2H-WSe_2_); Figure S4: (1), (2), (3), (4), (5), (6) show the HRTEM images, Selected Area Diffraction (SAED) pattern, and EDS spectrum of WSe_2_ slurry A. Similarly, (7), (8), (9), (10), (11), (12) show the HRTEM images, Selected Area Diffraction (SAED) pattern, and EDS spectrum of WSe_2_ slurry B and (13), (14), (15), (16), (17), (18), show the HRTEM images, Selected Area Diffraction (SAED) pattern, and EDS spectrum of WSe_2_ slurry C; Figure S5: AFM images of the slurries A as shown in (1) Height Profile: 4.5 ± 0.3 nm and (2) Height Profile: 4.6 ± 0.3 nm; B, as shown in (3) Height Profile: 3.1 ± 0.3 nm and (4) Height Profile: 4.3 ± 0.3 nm; C, as shown in (5) Height Profile: 4.4 ± 0.3 nm and (6) Height Profile: 5 ± 0.3 nm; Figure S6: XPS spectra for interpreting the surface states of WSe_2_ slurry-based coatings A (from (1) to (4)), B (from (5) to (8)), and C (from (9) to (14)); Table S1: Minimum and maximum R_s_ values measured for each WSe_2_-based cotton sample during humidity-sensing experiments; Figure S7. R_s_ trend of each sample following the humidity variation (from 30% to 90% RH) during the four exposure cycles; Table S2: Longest and shortest response times obtained for samples CO_A, CO_B, and CO_C during the four humidity exposure cycles; Table S3: Minimum and maximum R_s_ values measured for each WSe_2_-based cotton sample during temperature-sensing experiments; Figure S8: R_s_ trend of each sample following the temperature variation (from 20 to 60 °C) during the four exposure cycles; Table S4: Longest and shortest response times obtained for samples CO_A, CO_B, and CO_C during the four temperature exposure cycles.
