# Supplementary material for: Humidity- and Temperature-Sensing Properties of 2D-Layered Tungsten Di-Selenide (2H-WSe2) Electroconductive Coatings for Cotton-Based Smart Textiles
Source: Polymers (Basel). 2025 Mar 12;17(6):752. doi: 10.3390/polym17060752 (PMC11944947; doi:10.3390/polym17060752)
Supplement: Supplementary file 1 [file polymers-17-00752-s001.zip › polymers-3474894-supplementary.pdf]

*Supplementary Material*

# Humidity- and Temperature-Sensing Properties of 2D-Layered Tungsten Di-Selenide (2H-$WSe_2$) Electroconductive Coatings for Cotton-Based Smart Textiles

**Valentina Trovato [1,†,*], Rajashree Konar [2,3,†], Eti Teblum [2], Paolo Lazzaroni [1], Valerio Re [1], Giuseppe Rosace [1] and Gilbert Daniel Nessim [2,*]**

[1] Department of Engineering and Applied Sciences, University of Bergamo, Viale Marconi 5, 24044 Dalmine, Italy; paolo.lazzaroni@unibg.it (P.L.); valerio.re@unibg.it (V.R.); giuseppe.rosace@unibg.it (G.R.)
[2] Department of Chemistry and Institute of Nanotechnology & Advanced Materials (BINA), Bar-Ilan University, Ramat-Gan 5290002, Israel; rajashreekonar@gmail.com (R.K.); Eti.Teblum@biu.ac.il (E.T.)
[3] International Iberian Nanotechnology Laboratory, Av. Mte. José Veiga, 4715-330 Braga, Portugal; rajashreekonar@gmail.com (R.K.)
* Correspondence: valentina.trovato@unibg.it (V.T.); gilbert.nessim@biu.ac.il (G.D.N.)
† These authors contributed equally to this work.

**Keywords:** transition metal dichalcogenides; 2D materials; tungsten di-selenide; smart textiles; wearable sensors; textile finishing; electroconductive coatings; environmental monitoring

## 1.1. Bulk 2H-$WSe_2$ Synthesis in Ambient Pressure Chemical Vapor Deposition (APCVD)

The existing reports on the facile synthesis of 2D materials, exceptionally versatile materials like tungsten di-selenide in the CVD, highlight the challenges related to the scalability of the process. The procedure mentioned here demonstrates and confirms the extent of scalability of the $WSe_2$ synthesis on W foil using a simple fine-tuning of the ambient pressure CVD process parameters. The final yield of the 2D material grown on the W foil was engineered by introducing surface roughness via etching the W foil using 0.01 M HCl and careful control over the quantity of elemental Se used during the entire reaction. The synthesis procedure gave a uniform and homogenous few-layered film on ~2 cm x 6 cm of W foils (as shown below in Figure S1) that were chemically exfoliated using the liquid phase exfoliation (LPE) technique in an ultrasonication bath for the dispersions. The detailed growth mechanism of the pristine $WSe_2$ is already discussed. The resulting black 2H-$WSe_2$ powder was dried and used further to prepare mixtures with three different polymers.

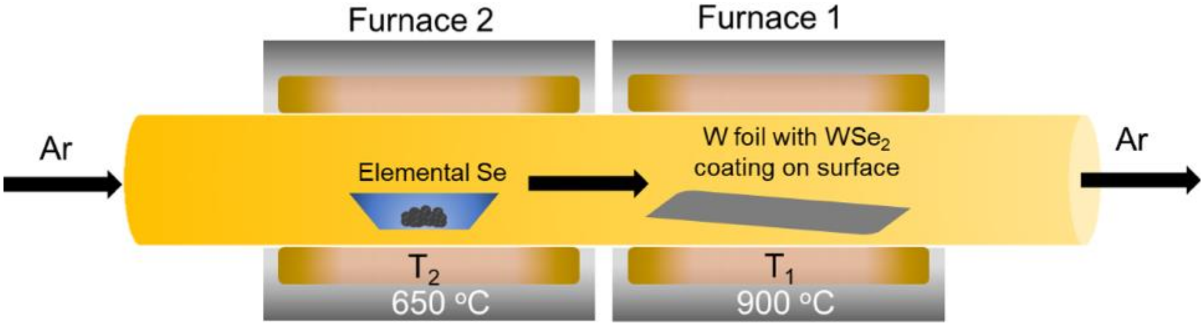


**Figure S1.** Schematic representation for tungsten di-selenide (2H-$WSe_2$) growth on W foil (using ambient pressure CVD), deposition of a few layers of 2H-$WSe_2$ on the surface of the W foil, and furnace setup.

For further investigations into the purity and quality of the final 2H-$WSe_2$ nanosheets, the bulk material was exfoliated using an ultrasonication bath with two different frequencies (37 and 80 kHz). Two low boiling point solvents, ethanol, and isopropanol, were also used as the liquid medium of choice for preparing the corresponding dispersions. The pristine exfoliated 2H-$WSe_2$ was then analyzed using high-resolution scanning electron microscopy (HRSEM), high-resolution transmission electron microscopy (HRTEM), and X-ray photoelectron spectroscopy (XPS).

## 1.2. Chemical and morphological characterization of pristine 2H-$WSe_2$ and 2H-$WSe_2$-based slurries

### *1.2.1. Microstructural analysis of pristine 2H-$WSe_2$*

The HRSEM image reported in Figure S2 (1) shows the bulk 2H-$WSe_2$ as grown on the W foil: the individual nanosheets of 2H-$WSe_2$ are arranged in a layered manner, indicating a 2D growth on the surface of the W foil. The exfoliation of the pristine 2H-$WSe_2$ nanosheets led to proper separation of the layers as revealed by the Atomic Force Microscopy (AFM) image in Figure S2 (2), which shows a height profile of about 1.1 ± 0.3 nm as drop-casted on a Si/$SiO_2$ substrate. The hexagonal nature of the $WSe_2$ (as signified by the 2H abbreviation in the beginning) is understood from the low-magnification and high-resolution

transmission microscopy (HRTEM) images, as shown in Figure S2 (3) and (4). The highlighted yellow portion in Figure S2 (5) shows the fringes in Figure S2 (4) belonging to the distinct (0002) plane ($d_{spacing}$= 0.683 nm) of 2H-$WSe_2$. A Selected Area Diffraction (SAED) pattern from the nanosheets in Figure S2 (5) corresponds to the planes related to 2H-$WSe_2$ as shown (008), (103), and (100), respectively. The nature of the point defects and intrinsic stacking fault type-I defects found in this exfoliated material was already investigated as per our previous studies on understanding the material properties. The fast Fourier transforms or FFTs in Figure S2 (4) indicate the typical honeycomb nature and the existence of the (001) fringe (d= 0.683 nm). The XRD of bulk 2H-$WSe_2$ is shown in Supplementary Figure S3.

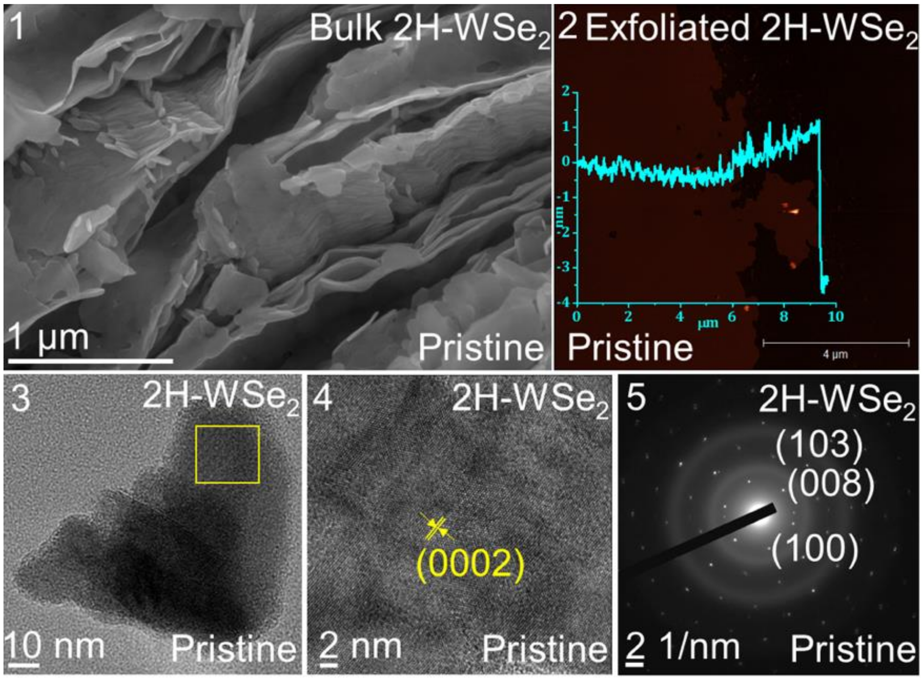


**Figure S2.** (1) High-Resolution Scanning Electron Microscopy (HRSEM) image of bulk tungsten di-selenide (2H-$WSe_2$); (2) Atomic Force Microscopy (AFM) measurement of exfoliated pristine 2H-$WSe_2$ nanosheets; (3) High-Resolution Transmission Electron Microscopy (HRTEM) image of exfoliated pristine 2H-$WSe_2$ nanosheets on Cu Grid; (4) High-Resolution Transmission Electron Microscopy (HRTEM) image of exfoliated pristine 2H-$WSe_2$; (5) Selected Area Diffraction (SAED) pattern from $WSe_2$ nanosheets.

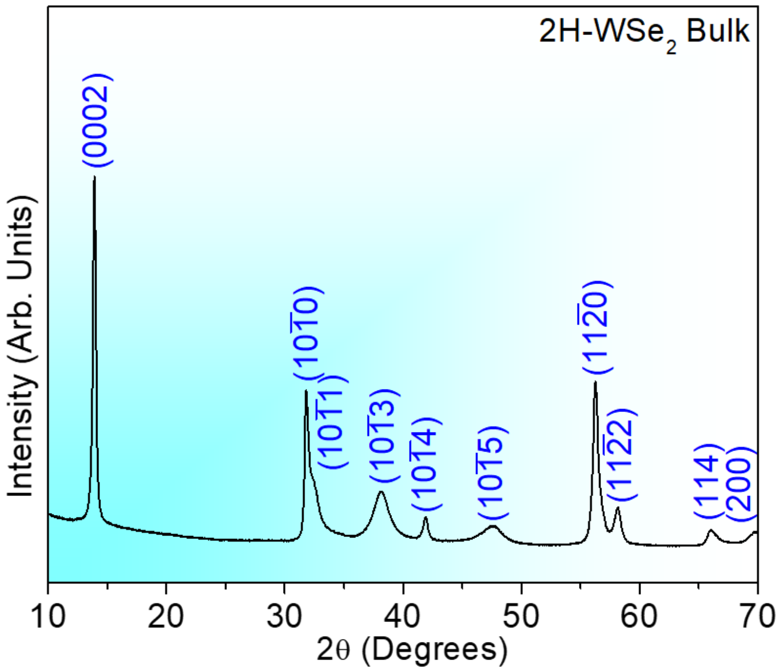


**Figure S3.** X-Ray Diffraction (XRD) pattern of bulk tungsten di-selenide (2H-WSe$_2$).

### 1.2.2. *Microstructural analysis of 2H-WSe$_2$-based slurries*

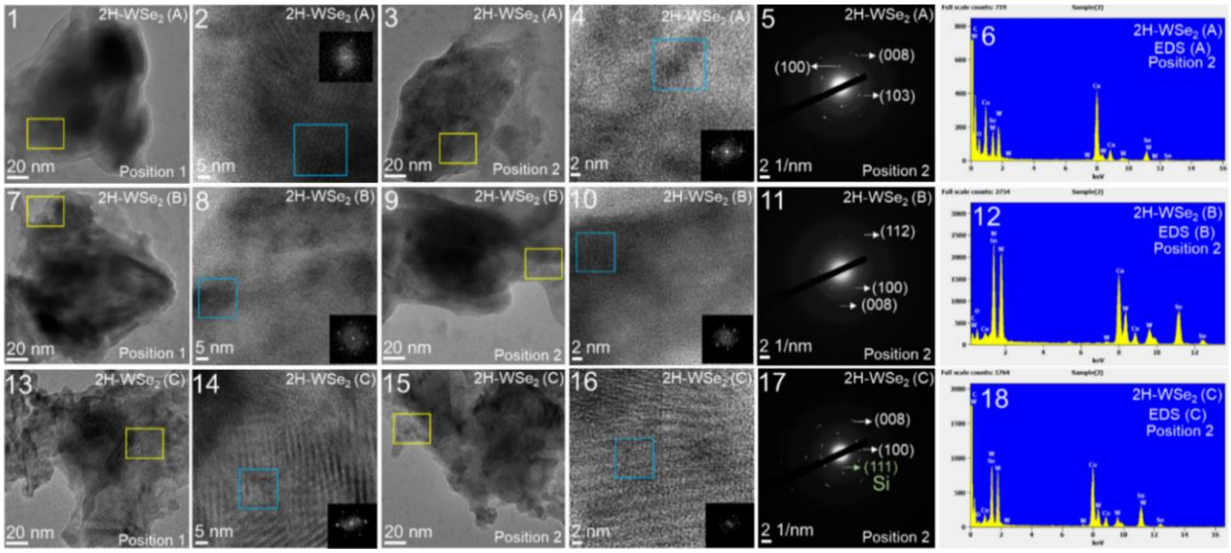


**Figure S4.** (1), (2), (3), (4), (5), (6) show the HRTEM images, Selected Area Diffraction (SAED) pattern and EDS spectrum of WSe$_2$ slurry A. Similarly, (7), (8), (9), (10), (11), (12) show the HRTEM images, Selected Area Diffraction (SAED) pattern and EDS spectrum of WSe$_2$ slurry B and (13), (14), (15), (16), (17), (18), show the HRTEM images, Selected Area Diffraction (SAED) pattern and EDS spectrum of WSe$_2$ slurry C.

For AFM measurements (Figure S5), the height profiles of the drop-cast samples were checked from two different positions on the substrates to detect and confirm homogeneity. Absolute isopropanol was the most compatible low-boiling point solvent for dispersing the slurries as scratched from the glass

slides. Per previous reports, a drop from the dispersions prepared at 37 kHz at100% power in an ultrasonic bath was placed on pre-cleaned $Si/SiO_2$ substrates.

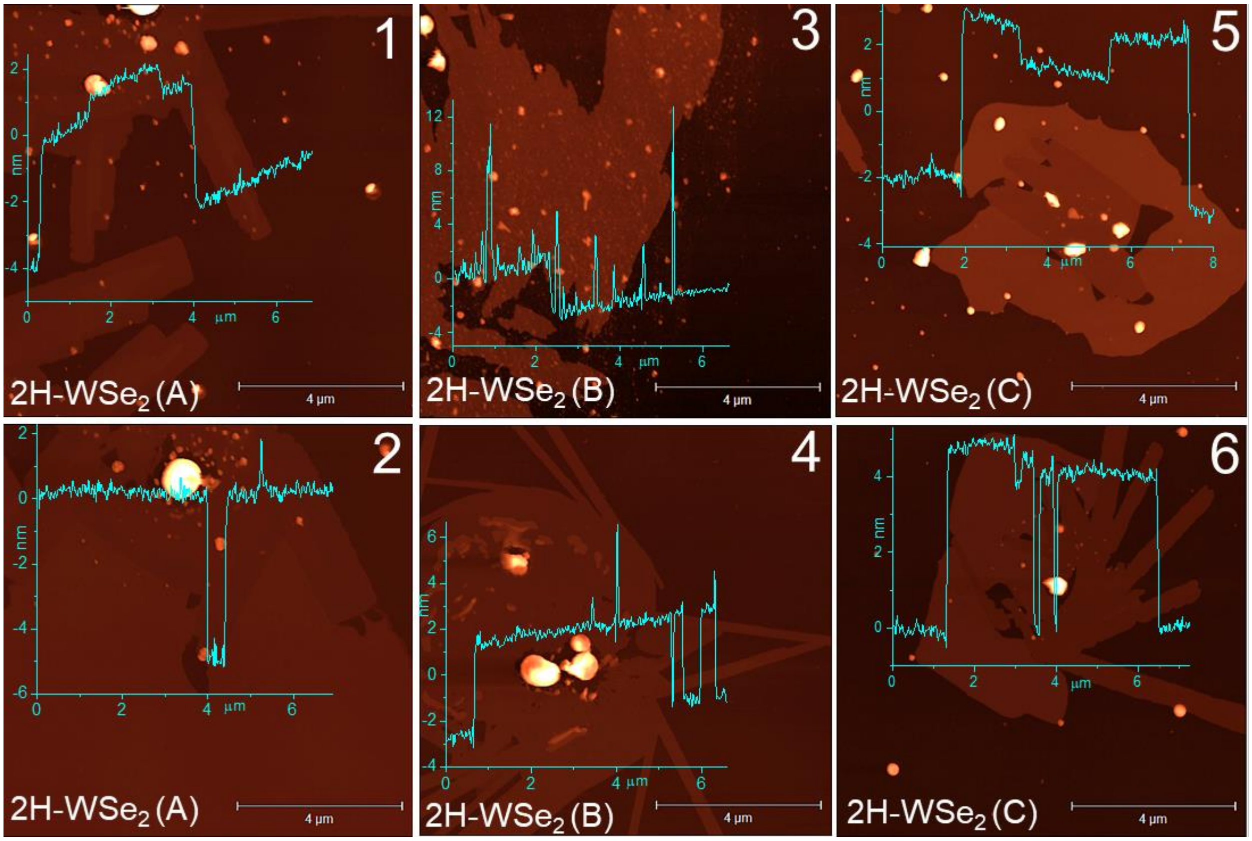


**Figure S5.** AFM images of the slurries A as shown in (1) Height Profile: 4.5 ± 0.3 nm and (2) Height Profile: 4.6 ± 0.3 nm; B, as shown in (3) Height Profile: 3.1 ± 0.3 nm and (4) Height Profile: 4.3 ± 0.3 nm; C, as shown in (5) Height Profile: 4.4 ± 0.3 nm and (6) Height Profile: 5 ± 0.3 nm.

XPS spectra are reported in Figure S6. The extent of W oxidation is presented in Figure S6 (1), which indicates the presence of W 4f core levels. The doublet observed at 32.6 eV and 34.8 eV corresponds to $4f_{7/2}$ and $4f_{5/2}$ lines, respectively, of $W^{4+}$ from pristine $WSe_2$. The other doublets at 36.2 eV and 38.1 eV correspond to the $4f_{7/2}$ and $4f_{5/2}$ lines of $W^{6+}$. The peak at 41.9 eV can be assigned to the W $5p_{3/2}$ core level of $WO_3$ due to slight oxidation from the exfoliation and mixing with the acrylic polymer in slurry A. The Se 3d core level spectrum of pristine $WSe_2$ (in the chemical state of $Se^{2-}$) exhibits a $3d_{5/2}$ and $3d_{3/2}$ doublet at 54.9 eV and 55.7 eV, as depicted in Figure S6 (2). Figures S6 (3) and (4) represent the chemical states of C and O, respectively, in coating A. The presence of C 1s core levels is assigned to C-H at 284.57 eV, C-C $sp^3$ at 285.11 eV, C-OH at 286.28 eV, C+O at 287.57 eV, and C-OOH at 289.1 eV. The O 1 s levels are assigned to $O_{I.}$ and $O_{I.I.}$ at 531.7 eV and 533.06 eV, respectively. Similarly, for slurry B, the W 4f core levels for $WSe_2$ nanosheets embedded in the polymer B matrix are at 32.6 eV and 34.8 eV, corresponding to $4f_{7/2}$ and $4f_{5/2}$ lines, respectively, thereby indicating $W^{4+}$ (Figure S6 (5)). The other doublets at 36.2 eV and 38.1 eV correspond to the $4f_{7/2}$ and $4f_{5/2}$ lines of $W^{6+}$, respectively (Figure S6 (5)). The Se 3d core level spectrum of pristine $WSe_2$ (in the chemical state of $Se^{2-}$) exhibits a $3d_{5/2}$ and $3d_{3/2}$ doublet at 54.9 eV and 55.7 eV, as depicted in Figure S6 (6). The presence of C 1s core levels is assigned to C-H at 284.8 eV, C-C $sp^3$ at 285.3 eV, C-OH at 286.2 eV, C = O at 286.8 eV, and C-OOH at 288.8 eV. The O 1s levels are assigned to $O_{I.}$ and $O_{I.I.}$ at 531.7 eV and 533.06 eV,

respectively. Figure S6 (9) for Slurry C presents a similar spectrum of W 4f core levels. The doublet observed at 32.6 eV and 34.8 eV corresponds to $4f_{7/2}$ and $4f_{5/2}$ lines, respectively of $W^{4+}$ from pristine $WSe_2$. The other doublets at 36.2 eV and 38.1 eV correspond to the $4f_{7/2}$ and $4f_{5/2}$ lines, respectively, of $W^{6+}$ (Figure S6 (9)). The Se 3d core level spectrum of pristine $WSe_2$ (in the chemical state of $Se^{2-}$) exhibits a $3d_{5/2}$ and $3d_{3/2}$ doublet at 54.9 eV and 55.7 eV, as depicted in Figure S6 (10). The presence of C 1s core levels is assigned to C-H at 284.5 eV, C-C $sp^3$ at 285.1 eV, C-OH at 286.3 eV, and C-OOH at 288.9 eV. The O 1s levels are assigned to $O_{.I.}$ and $O_{.I.I.}$ at 531.7 eV and 533.06 eV, respectively. Additionally, Si 1s (2p $_{3/2}$ at 102.26 eV and $2p_{1/2}$ at 102.69 eV) and N 1s ($sp^3$ C-N and $sp^2$ C-N at 102.25 eV and 102.68 eV, respectively) are also recorded in Figures S6 (13) and (14).

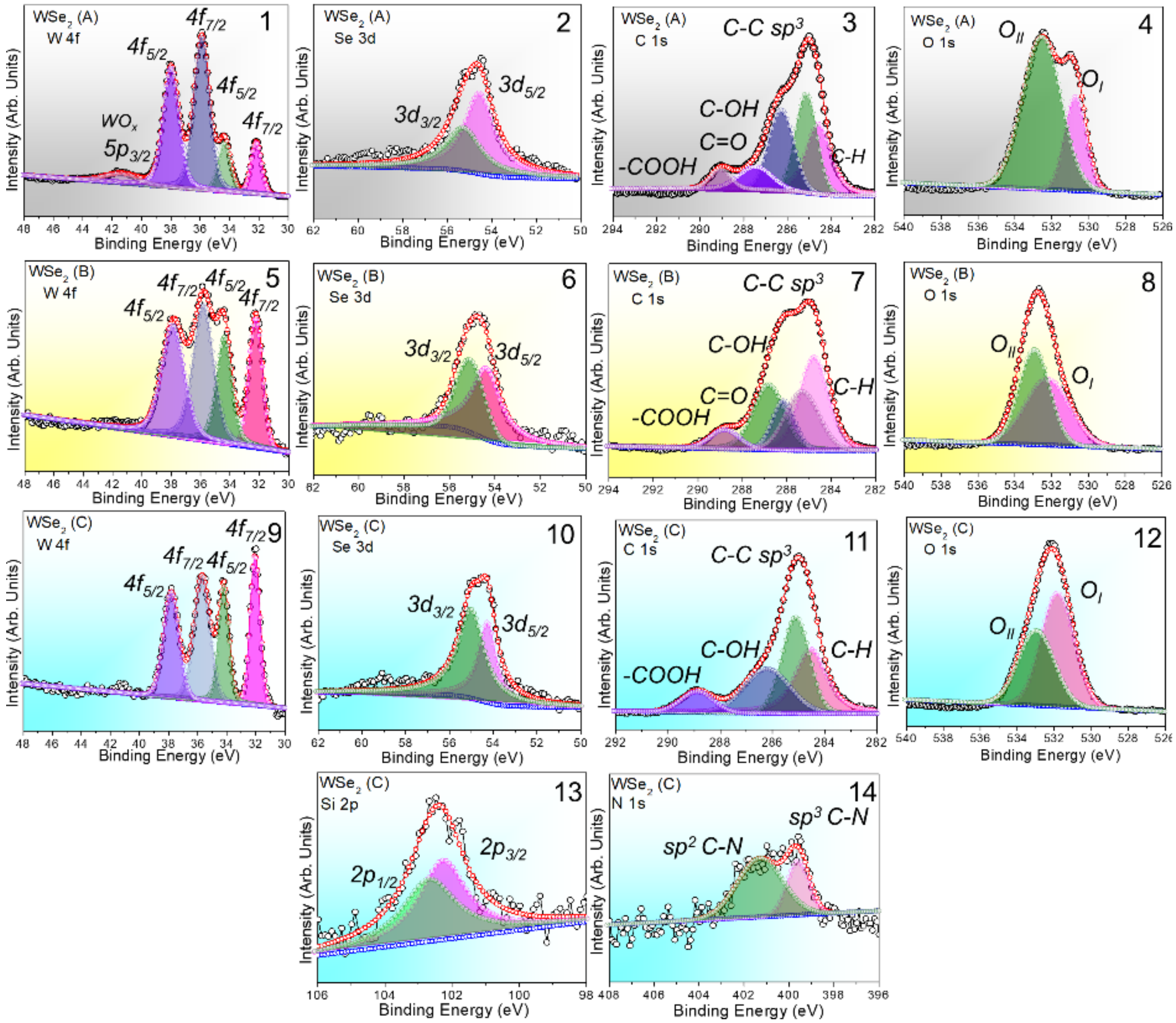


**Figure S6.** XPS spectra for interpreting the surface states of $WSe_2$ slurry-based coatings A (from (1) to (4)), B (from (5) to (8)), and C (from (9) to (14)).

## 1.3. Humidity and temperature sensing of $WSe_2$-based cotton fabrics

2H-$WSe_2$-treated cotton samples characterized before experiments showed at room conditions (25 ±0.04 °C and 50 ±0.44% RH) average $R_s$ values equal to $1.57 \cdot 10^9 \pm 4.83 \cdot 10^7$ Ω sq$^{-1}$, $2.87 \cdot 10^8 \pm 1.16 \cdot 10^7$ Ω sq$^{-1}$, and $1.14 \cdot 10^9 \pm 2.53 \cdot 10^7$ Ω sq$^{-1}$ for samples A, B and C, respectively. These $R_s$ values made the treated fabrics too resistive for standard discrete component electronics to deal with currents provided by them. Moreover, considering the obtained $R_s$ values of the coated cotton strips and tens of voltage as the reference power supply, one needs to be able to detect currents down to tens of nA to be able to operate the strip.

### *1.3.1 Humidity-sensing performance of 2D-doped cotton fabrics*

**Table S1.** Minimum and maximum $R_s$ values measured for each $WSe_2$-based cotton sample during humidity sensing experiments.

| Sample | Min $R_s$ (Ω sq$^{-1}$) | Max $R_s$ (Ω sq$^{-1}$) |
|---|---|---|
| CO_A | $7.34 \cdot 10^5 \pm 5.57 \cdot 10^4$ | $4.87 \cdot 10^9 \pm 6.01 \cdot 10^7$ |
| CO_B | $3.83 \cdot 10^5 \pm 2.88 \cdot 10^4$ | $7.03 \cdot 10^8 \pm 1.84 \cdot 10^7$ |
| CO_C | $1.72 \cdot 10^7 \pm 2.43 \cdot 10^6$ | $2.03 \cdot 10^9 \pm 9.09 \cdot 10^6$ |

The results were proven to be highly repeatable for samples CO_B and CO_C with a deviation of less than ± 10% for all RH%, while $R_s$ values of sample CO_A revealed the best repeatability between the second and the fourth exposure cycles (Figure S7).

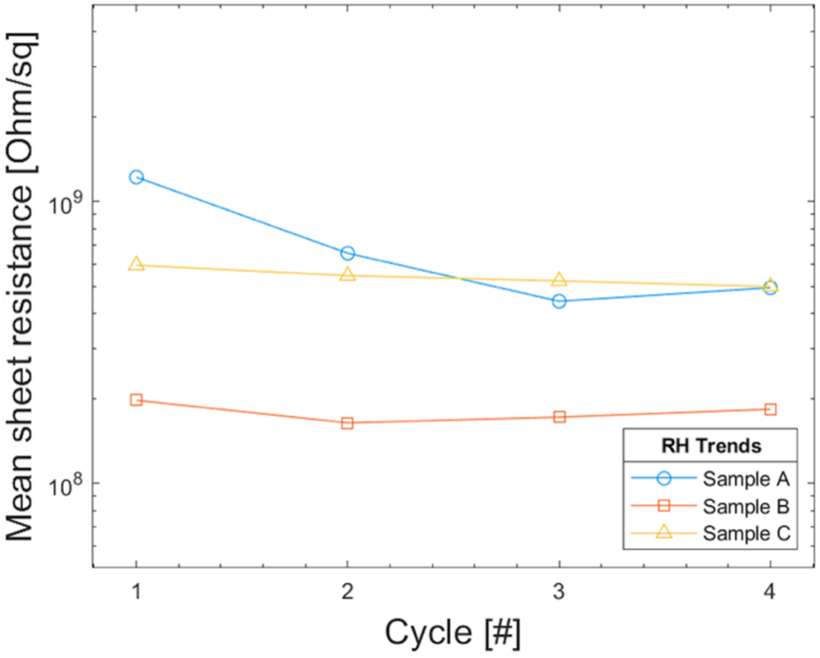


**Figure S7.** $R_s$ trend of each sample following the humidity variation (from 30% to 90% RH) during the four exposure cycles.

For all samples, the shortest and the longest response time was established for each humidity exposure cycle (Table S2). The three samples revealed short response times (between 4 and 14 minutes) for

low humidity levels (30%–40% RH) and longer response times (≥30 minutes) for high moisture levels (higher than 70% RH). These findings can be ascribed to the water uptake of the coating, which is higher for high moisture levels than lower ones.

**Table S2.** Longest and shortest response times obtained for samples CO_A, CO_B and CO_C during the four humidity exposure cycles.

| | | Longest response time (min) | | Shortest response time (min) | |
|---|---|---|---|---|---|
| **Sample** | **Cycle** | **In the range 30–90% RH (%RH interval)** | **In the range 90–30% RH (%RH interval)** | **In the range 30–90% RH (%RH interval)** | **In the range 90–30% RH (%RH interval)** |
| CO_A | 1 | ≥30 (70–75%) | 27 (70–60%) | 10 (30–40%) | 6 (90–85%) |
| | 2 | ≥30 (85–90%) | ≥30 (70–60%) | 8 (30–40%) | 12 (90–85%) |
| | 3 | 29 (85–90%) | ≥30 (60–50%) | 6 (30–40%) | 13 (90–85%) |
| | 4 | ≥30 (85–90%) | 25 (70–60%) | 14 (30–40%) | 12 (90–85%) |
| CO_B | 1 | ≥30 (85–90%) | ≥30 (60–50%) | 7 (30–40%) | 14 (90–85%) |
| | 2 | ≥30 (85–90%) | ≥30 (60–50%) | 12 (30–40%) | 12 (90–85%) |
| | 3 | ≥30 (85–90%) | ≥30 (70–60%) | 6 (30–40%) | 13 (40–30%) |
| | 4 | ≥30 (85–90%) | ≥30 (70–60%) | 9 (30–40%) | 12 (90–85%) |
| CO_C | 1 | ≥30 (85–90%) | ≥30 (70–60%) | 8 (40–50%) | 7 (90–85%) |
| | 2 | | ≥30 (60–50%) | 6 (30–40%) | 7 (90–85%) |
| | 3 | ≥30 (80–85%) | ≥30 (60–50%) | 4 (30–40%) | 8 (40–30%) |
| | 4 | ≥30 (85–90%) | 25 (60–50%) | 4 (30–40%) | 7 (40–30%) |

### *1.3.2. Temperature-sensing performance of 2D-$WSe_2$-based fabrics*

**Table S3.** Minimum and maximum $R_s$ values measured for each $WSe_2$-based cotton sample during temperature sensing experiments.

| Sample | Min $R_s$ ($\Omega$ sq$^{-1}$) | Max $R_s$ ($\Omega$ sq$^{-1}$) |
|---|---|---|
| CO_A | ~ $1.5 \cdot 10^8$ | ~ $9.0 \cdot 10^9$ |
| CO_B | ~ $3.0 \cdot 10^7$ | ~ $8.0 \cdot 10^8$ |
| CO_C | ~ $2.0 \cdot 10^8$ | ~ $2.0 \cdot 10^9$ |

The results were highly repeatable, with a deviation of less than ± 10% for all temperature values, as reported in Figure S8.

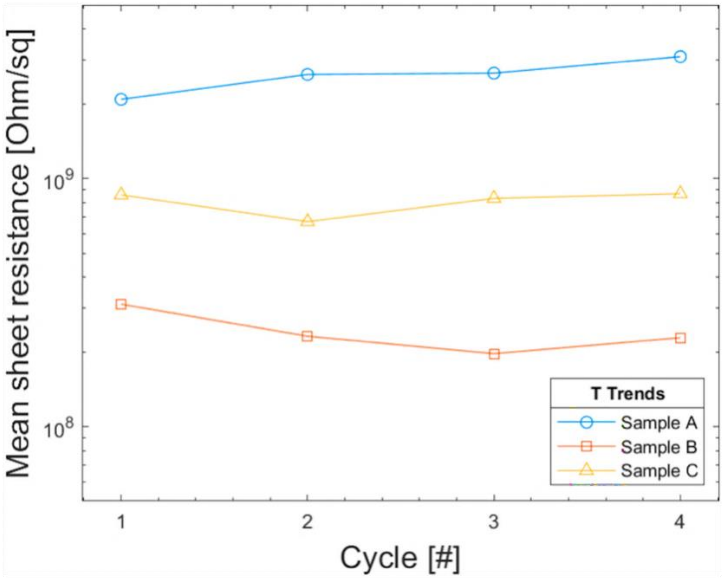


**Figure S8.** $R_s$ trend of each sample following the temperature variation (from 20 to 60 °C) during the four exposure cycles.

As evident from Table S4, all samples revealed shorter response times for temperatures lower than 30°C, while for higher temperatures, the sensors had longer response times.

**Table S4.** Longest and shortest response times obtained for samples CO_A, CO_B and CO_C during the four temperature exposure cycles.

| | | Longest response time (hours) | | Shortest response time (hours) | |
|---|---|---|---|---|---|
| **Sample** | **Cycle** | **In the range 20–60 °C (°C interval)** | **In the range 60–20 °C (°C interval)** | **In the range 20–60 °C (°C interval)** | **In the range 60–20 °C (°C interval)** |
| CO_A | 1 | ≥2.5 (50–60 °C) | 1.6 (60–50 °C) | 1.8 (20–25 °C) | 0.8 (25–20 °C) |
| | 2 | ≥2.5 (50–60 °C) | 1.8 (60–50 °C) | 1.7 (20–25 °C) | 0.9 (25–20 °C) |
| | 3 | ≥2.5 (50–60 °C) | 2 (60–50 °C) | 1.8 (20–25 °C) | 1.2 (25–20 °C) |
| | 4 | ≥2.5 (50–60 °C) | 2 (60–50 °C) | 2 (20–25 °C) | 1.4 (25–20 °C) |
| CO_B | 1 | ≥2.5 (50–60 °C) | 2.2 (60–50 °C) | 1.7 (20–25 °C) | 0.6 (25–20 °C) |
| | 2 | ≥2.5 (50–60 °C) | 2.2 (60–50 °C) | 2.3 (20– 25 °C) | 0.8 (25–20 °C) |
| | 3 | ≥2.5 (50–60 °C) | 2 (60–50 °C) | 1.4 (20–25 °C) | 1.2 (25–20 °C) |
| | 4 | ≥2.5 (50–60 °C) | 2 (60–50 °C) | 1.4 (20–25 °C) | 1.2 (25–20 °C) |
| CO_C | 1 | 1.5 (50–60 °C) | 0.5 (60–50 °C) | 0.2 (20–25 °C) | 0.4 (30–25 °C) |
| | 2 | 2 (50–60 °C) | 1 (60–50 °C) | 1.7 (25–30 °C) | 0.6 (30–25 °C) |
| | 3 | 1.7 (50–60 °C) | 1.3 (60–50 °C) | 0.7 (20–25 °C) | 0.4 (30–25 °C) |
| | 4 | 1.7 (50–60 °C) | 1.3 (60–50 °C) | 0.6 (20–25 °C) | 0.7 (30–25 °C) |

In particular, the response times for lower temperatures are overall significantly longer when the temperature was increased from 20 to 60 °C than when it was returned from 60 to 20 °C for the same range

of temperatures, with the exception made for cycles 1, 3 and 4 of sample C. Conversely, response times measured near 60°C behave opposite.

Also, CO_A and CO_B have response times longer than 2.5 hours to provide a stable response, regardless of the number of exposure cycles. CO_C seems to be faster with the longest response time of 2 hours (cycle 2). The parallelism can be maintained for what concerns response times when temperatures are returned from 60 to 20 °C. In this regard, CO_A and CO_B showed a maximum response time of 2 and 2.2 hours respectively, while CO_C maximum response time is around 1.3 hours.
